# Biofortification of Potato and Carrot With Iodine by Applying Different Soils and Irrigation With Iodine-Containing Water

**DOI:** 10.3389/fpls.2020.593047

**Published:** 2020-12-09

**Authors:** Péter Dobosy, Anett Endrédi, Sirat Sandil, Viktória Vetési, Márk Rékási, Tünde Takács, Gyula Záray

**Affiliations:** ^1^MTA Centre for Ecological Research, Danube Research Institute, Budapest, Hungary; ^2^GINOP Evolutionary Systems Research Group, MTA Centre for Ecological Research, Tihany, Hungary; ^3^Cooperative Research Centre of Environmental Sciences, Eötvös Loránd University, Budapest, Hungary; ^4^MTA Centre for Agricultural Research, Institute for Soil Sciences and Agricultural Chemistry, Budapest, Hungary

**Keywords:** mineral nutrition, soil type, iodine uptake, *Solanum tuberosum* L., *Daucus carota* L.

## Abstract

Accumulation of iodine by potato (*Solanum tuberosum* L.) and carrot (*Daucus carota* L. var. *sativus*) plants cultivated on different soils (sand, sandy silt, and silt) using irrigation water containing iodine at concentrations of 0.1 and 0.5 mg/L was investigated. In the edible organs of potato and carrot control plants grown on sand, sandy silt, and silt soils, the iodine concentrations were 0.15, 0.17, and 0.20 mg/kg (potato) and 0.012, 0.012, and 0.013 mg/kg (carrot); after the treatment by applying 0.5 mg/L iodine dosage, the iodine concentrations were 0.21, 0.19, 0.27 mg/kg (potato) and 3.5, 3.7, 3.0 mg/kg (carrot), respectively. Although the iodine treatment had no significant effect on the biomass production of these plants, in potato tubers, it resulted in higher Fe and lower Mg and P concentrations, whereas no similar trend was observable in carrot roots. The accumulation of Mn, Cu, Zn, and B in the edible part of both plants was not influenced by the iodine treatment. The soil properties did not have a significant impact on biomass production under the same environmental conditions. The concentration and the distribution of iodine in both plants were slightly modified by the growing medium; however, the photosynthetic efficiency and the chlorophyll content index of potato plants cultivated in silt soil increased significantly. Potato plant was not suitable for biofortification with iodine, while considering the iodine concentration and the moisture content of carrot roots, it can be calculated that consuming 100 g fresh carrot would cover about 38% of the daily iodine intake requirement for an average adult person.

## Introduction

Iodine is an essential micronutrient for human health, having a unique role in the regulation of metabolic processes such as synthesis of thyroid hormones (triiodothyronine and thyroxine), which are involved in the synthesis of sugars, nucleic acids, and proteins (Velasco et al., 2018). The recommended dietary allowance for iodine ranges between 90 and 270 μg: 90 μg—young children (1–8 years), 120 μg—older children (9–13 years), 150 μg—adults, and 220–270 μg—pregnant and lactating woman. Iodine deficiency is a widespread problem in the world, which leads to iodine deficiency disorders (IDDs) like goiter, hypothyroidism, psychomotor defects, hearing/speech impairment, and developmental delay (Hays et al., 2018; Krzepiłko et al., 2019). Iodine supplementation and elimination of IDDs are based on the application of iodized table salts; however, 90% of the iodine content of salt can be lost due to low iodine stability and the losses during the production steps (packaging, transportation, and processing) (Winger et al., 2008). Furthermore, many countries have adopted the regulations of the World Health Organization (WHO) for the reduction of salt consumption in order to prevent cardiovascular diseases and hypertension (Kurosad et al., 2005). Iodine biofortification of vegetables has been proven to be an alternative and cost-effective way to provide iodine in the human diet. Several strategies in agriculture (e.g., hydroponics, pot, and field experiments) have been developed to increase iodine content in the edible part of plants as demonstrated in lettuce (Blasco et al., 2008, 2012; Hong et al., 2008; Voogt et al., 2010; Dobosy et al., 2020a), spinach (Dai et al., 2006; Weng et al., 2008b; Smoleń et al., 2016a), bean (Dobosy et al., 2020a), pakchoi (Hong et al., 2009), cabbage (Weng et al., 2008a; Mao et al., 2014; Ojok et al., 2019; Dobosy et al., 2020b), Chinese cabbage (Hong et al., 2008), tomato (Hong et al., 2008; Caffagni et al., 2011; Landini et al., 2011; Kiferle et al., 2013; Smoleń et al., 2015; Halka et al., 2020; Dobosy et al., 2020b), rice (Kato et al., 2013), strawberry (Li et al., 2016), cucumber (Voogt et al., 2014), kohlrabi (Golob et al., 2020), celery (Hong et al., 2009), and radish (Kato et al., 2013; Lawson et al., 2015). On the basis of the literature data, the following statements can be summarized: (1) the major species of iodine like organic-iodine compounds, iodate (IO_3_^–^), and iodide (I^–^) ions in the soil and volatile iodine forms (molecular iodine and methyl iodide) in the atmosphere can be efficiently taken up by the roots and leaves (dominant); (2) due to the relatively large leaf surface in leafy vegetables (spinach, lettuce, cabbage, Chinese cabbage), iodine can be taken up more rapidly and effectively from the atmosphere; (3) iodine moves mainly by xylematic routes; therefore, the iodine concentration of a plant decreases from the root to the fruit, i.e., iodine is stored better in vegetative plant tissues than in fruits; however, the phloematic way has been also reported for some plants, e.g., tomato, lettuce; (4) as compared to iodate, accumulation of iodide by the roots and translocation to the different upper parts (e.g., stem, leaf, fruit) are more efficient; and (5) over a certain iodine concentration (e.g., 1 mg/L in hydroponic solution or 25 mg/kg using fertilizer), toxic effect in plants can be observed, resulting in reduced biomass production.

The iodine concentration of different soil types occur in the range of < 0.1–150 mg/kg and is strongly dependent on the characteristic of soils (e.g., pH, microbial activity, organic and inorganic components). Some studies have reported that microbes in the root promote reductive conditions, thus transforming iodate to iodine with the help of specific reductase enzymes. Organic (e.g., humic and fulvic acids) and inorganic components (e.g., metal oxides) play a dominant role in iodine transport in soils by binding a considerable proportion of iodine, thereby increasing the fixation and decreasing the bioavailability of iodine for plants (Medrano-Macías et al., 2016; Gonzali et al., 2017). Potato and carrot are among the most popular cultivated vegetables in the world, and in 2018, the total production was 368 and 40 million tons in 17.5 and 1.1 million hectares, respectively. These vegetables could therefore be good target plants for biofortification with iodine (FAO, 2019). In spite of the mass production of these plants over the world, only a few biofortification experiments with iodine have focused on potato and carrot plants using various fertilizers and technologies.

In this paper, the iodine accumulation of potato and carrot plants cultivated in different soils (sand, sandy silt, and silt) and irrigated with iodine-containing water at concentrations of 0.1 and 0.5 mg/L (as potassium iodide) was investigated. In addition, the effect of iodine on photosynthetic efficiency and plant growth and the accumulation of selected macro- and microelements (P, Mg, Mn, Fe, Cu, Zn, B) in the edible parts of the plants were also studied.

## Materials and Methods

### Chemicals and Reagents

All chemicals applied for sample preparation and elemental analyses were of analytical grade. For the preparation and the dilution of standard solutions, ultra-pure water (18 MΩ cm^–1^) was taken from a WasserLab Autwomatic unit (Labsystem Ltd., Budapest, Hungary). Iodine standard solutions were prepared using solid KIO_3_ (Sigma Aldrich Ltd., Missouri, United States), and for determination of selected macro- and microelements (P, Mg, Mn, Fe, Cu, Zn, and B), a multi-element standard solution (Sigma Aldrich Ltd., Missouri, United States) was applied. The accuracy of the analytical measurements was verified by using NIST SRM 1573a tomato leaf (National Institute of Standards and Technology, Gaithersburg, United States) certified reference material.

### Characterization of Soils

The three investigated top (0–20 cm) soils from Hungary were sand [Mollic Umbrisol (Arenic) from Őrbottyán], silty sand (Luvic Calcic Phaeozem from Gödöllő), and silt (Calcic Chernozem from Hatvan). The soil properties are shown in Table 1. The pH was determined according to the Hungarian standard method by applying 1:2.5 soil/water solution after mixing for 12 h. The CaCO_3_ content was measured using Scheibler gas-volumetric method (MSZ-08-0206/2, 1978). Organic matter (OM) content was determined using modified Walkley–Black method (MSZ-08-0452, 1980). Bioavailable P and K concentrations were measured following ammonium-acetate lactate extraction (AL-P_2_O_5_ and AL-K_2_O) (Egnér et al., 1960). Total nitrogen content was measured by the Kjeldahl method (ISO 11261, 1995) and NH_4_–N and NO_3_–N concentrations were determined from KCl (Sigma Aldrich Ltd., Missouri, United States) extracts (MSZ 20135, 1999). The cation exchange capacity (CEC) was measured using modified Mehlich method (MSZ-08-0215, 1978), and iodine concentrations were determined by inductively coupled plasma mass spectrometry (ICP-MS) (Plasma Quant MS Elite, Analytik Jena, Jena Germany) following microwave-assisted aqua regia digestion of soil samples using a TopWave equipment (Analytik Jena, Jena, Germany), applying In as internal standard at a concentration of 20 μg/L.

**TABLE 1:** Physico-chemical parameters of soils.

Parameters	Sand	Sandy silt	Silt
pH—H_2_O	7.96	6.83	7.34
OM (w/w%)	0.91	1.24	2.12
CaCO_3_ (w/w%)	1.45	0.08	0.20
Total-N (w/w%)	0.064	0.092	0.135
NH_4_–N (mg/kg)	1.4	2.3	3.9
NO_3_–N (mg/kg)	4.7	2.3	14.2
Al–K_2_O (mg/kg)	74	174	176
Al–P_2_O_5_ (mg/kg)	131	238	81
Cation exchange capacity (Na meq/100 g)	9	17	37
Iodine concentration (mg/kg)	1.2	1.9	1.2

### Plant Cultivation

Potato tubers (*Solanum tuberosum* L. cv. Balatoni rózsa) and carrot seeds (*Daucus carota* L. var. *sativus* cv. Nantes-2) were germinated and planted for 3 weeks under controlled climatic conditions (16/8 h photoperiod and a temperature setting of 26/16°C, 600 μmol/m^2^/s photon flux density) in a commercial growth medium (VEGASCA Bio; Florasca). The effect of irrigation was investigated in a pot experiment. The experiment was performed in an open greenhouse at the Experiment Station of Center of Agricultural Research in Őrbottyán, Hungary, using 10-L pots with four holes (Ø 0.5 cm) at the bottom so that the leaching water could flow out. The bottom of the pot was filled with gravel (4–8 mm) at a 1 cm layer. The gravel layer was covered with a fine synthetic fiber fabric on which the applied soil was layered in 10 kg volume. Irrigation has been carried out with an automatic irrigation system. The irrigation water has been delivered using individual drip stakes placed in each pot. The daily volume of irrigated water has been set by the water requirements of the plants. A monitoring system measured soil moisture content at 10 cm depth in every hour (Sensor: Decagon EC-5). The irrigation system delivered the set amount of water every day at 7 a.m.

After transplantation, the reared seedlings (one potato plant/pot and three carrot plants/pot) were irrigated with drinking water for 3 weeks. The 76-day-old potato and 106-day-old carrot plants were harvested. The potato plants had not reached the end of their photosynthetically active period. The *in situ* measurements were taken on the youngest and green adult leaves.

During the growing period, the plants (including control) were watered weekly with Hoagland solution (150 ml per pot) by hand. The irrigation with iodine solution started 3 weeks after planting. In the experiments, two types of plants were grown on three different soils applying three treatment levels (control + two iodine dosages); the applied iodine concentration in the irrigation water amounted to 0.1 and 0.5 mg/L, and in all cases, three replicates were investigated. The iodine solutions were made of KI diluted with drinking water. The water was stored in 0.5 m^3^ tanks (separate tanks for each irrigation solution) before application to reduce the chlorine concentration. The element concentrations in the tanks were monitored to provide constant concentrations. The details of the irrigation can be found in Table 2.

**TABLE 2:** Growing, cultivation, and irrigation parameters of the experimental setup.

Parameters	Potato	Carrot
Growing period	24 May 2018–17 July 2018	11 April 2019–04 July 2019
Length of growing period (days)	55	85
Total irrigation (L/pot)	19.5	17.5
I load in 0.1 mg/L treatment (mg/pot)	1.06	1.11
I load in 0.5 mg/L treatment (mg/pot)	5.30	5.55

The experimental area received natural light in a greenhouse, for which climate data were continuously monitored during the growth period. The measured parameters of the sensors in the greenhouse are shown in Table 3. During the growing period, pesticide (Decis, Bayer) was applied on the carrot when it was necessary.

**TABLE 3:** Greenhouse parameters for the growing period of potato and carrot.

Parameters	Potato	Carrot
Daytime average temperature (°C)	25.63.5	22.46.6
Nighttime average temperature (°C)	18.12.3	14.65.1
Photosynthetically active radiation (W/m^2^)	240107	15660
Air humidity (%)	69.723.3	72.524.9
Soil moisture (v/v%)	226	226

### Photosynthetic Efficiency and Chlorophyll Content Index

The photosynthetic efficiency and the chlorophyll content index (CCI) of potato leaves were determined at the harvesting stage. In the case of carrot plants, these parameters cannot be measured *in situ* due to the structure of the leaf organs. The photosynthetic efficiency was characterized by measuring the quantum efficiency (Fv/Fm) of photosystem (PS) II using an Os30p + hand-held chlorophyll fluorometer (Opti-Sciences, Hudson, United States). To indicate the potential stress of potato caused by iodine treatment, the Fv/Fm ratios were calculated, where Fv = variable fluorescence level from dark-adapted leaves and Fm = maximal fluorescence level from dark-adapted leaves. Before the determination of Fv/Fm ratios, the potato leaves were dark-adapted for 15 min. The CCI of leaves was determined by applying a CCM-200 plus Chlorophyll Content Meter (Opti-Sciences, Hudson, United States). The *in situ* measurements were taken on the youngest adult leaves. The CCI values were calculated from the average of three measurements per plant.

### Sample Preparation and Elemental Analysis

The plants were harvested and washed with deionized water, and then different plant parts were separated [root, aerial part (stem + leaf), tuber]. Potato roots and the aerial parts of both plants were dried at 40°C in a laboratory dryer for 2 days, while carrot roots and potato tubers were freeze-dried at −70°C, 200 Pa, for 72 h, and then the dry mass of the plant organs was determined. For homogenization of these dried samples, a household blending machine, equipped with plastic housing and a stainless-steel blend, was used. The dried and homogenized samples were mineralized in a microwave-assisted acid digestion system (TopWave, Analytik Jena, Germany). A total of 400–500 mg plant samples was digested in a mixture of 7 ml 67% HNO_3_ (VWR International, Pennsylvania, United States) and 3 ml 30% H_2_O_2_ (VWR International, Pennsylvania, United States). After digestion, the internal standards (Sc, Y, In) were added to the solutions and filled up to 15 ml with deionized water. The concentration of iodine and macro- and microelements was measured by ICP-MS. To check the accuracy of the analytical procedure, the recovery values for the investigated eight elements were determined by analyzing the NIST tomato leaf SRM sample, and the results were between 92 and 107%.

### Statistical Evaluation

Data visualization and statistical analysis were made with R statistical software (R Core Team, 2019). The line plots visualizing the mean and the standard deviations (SD) of the data were made with the “ggpubr” package (Kassambara, 2019). Linear models were used to compare the impact of treatment dosages, soil types, their interactions on the average photosynthetic efficiency, plant growth, iodine and micro- and macroelement content of the plants, and iodine distribution among plant parts. *Post hoc* pair-wise comparisons were made by Tukey test, using the “glht” function of the “multcomp” package (Hothorn et al., 2008).

## Results

### Photosynthetic Efficiency and Chlorophyll Content Index

The photosynthetic efficiency of photosystem II (dark-adapted Fv/Fm ratio) and the chlorophyll content index (CCI) were determined for potato leaves, and the data measured *in situ* are presented in Table 4. The Fv/Fm changed in the range of 0.667–0.747, the soil type did not affect it (*p* > 0.089), and the iodine treatment also had no significant effect on it (*p* > 0.36), independently from the soil type. In contrast, the CCI values showed significant differences between the soils: plants on sandy and sandy silt soils showed significantly lower CCI values than plants on silt soil (*p* < 0.018). The iodine treatment resulted in a marginally significant, positive effect on the CCI of potato cultivated on sand (*p* = 0.059) and silt soils (*p* = 0.065), while a moderate but non-significant reduction of CCI values was observed in the case of sandy silt soil (*p* > 0.50). In summary, the iodine treatment had no significant impact, neither on Fv/Fm nor on CCI values, but for potato plants cultivated in silt soils, these parameters (especially CCI) were higher than in sand and sandy silt soils.

**TABLE 4:** Average photosynthetic efficiency and chlorophyll content index of potato leaf organs cultivated in three different soils and irrigated with water containing iodine.

Photosynthetic efficiency and chlorophyll content index of leaves (*n* = 3; RSD%)			

	Iodine concentration in irrigation water	Fv/Fm	CCI
Sand	Control	0.667(4)^a^	11.1(9)^a^
	0.1 mg/L	0.716(4)^a^	15.4(22)^a^
	0.5 mg/L	0.691(8)^a^	16.5(10)^a^
Sandy silt	Control	0.734(6)^a^	13.1(17)^a^
	0.1 mg/L	0.693(1)^a^	11.3(8)^a^
	0.5 mg/L	0.728(6)^a^	11.6(17)^a^
Silt	Control	0.726(6)^a^	19.0(11)^a^
	0.1 mg/L	0.747(4)^a^	25.4(5)^a^
	0.5 mg/L	0.731(5)^a^	22.3(19)^a^

*Different letters indicate significant differences (p < 0.05, Tukey’s test) within the soil types.*

### Effect of Iodine on Plant Growth

The dry mass values of plant parts of the control and the iodine-treated potato and carrot plants are presented in Table 5. On comparing the effect of soil type on biomass production without any treatment, the differences in the dry mass of the root and tuber of potato plants were negligible (*p* > 0.3), while the aerial parts showed a higher, but also not significant, difference (*p* > 0.3), achieving the lowest and the highest masses on sandy silt and silt soils, respectively. In the case of carrot, a different pattern was observed: both the dry mass of root and aerial parts increased in the order of silt < sand < sandy silt; however, only the roots showed a significantly higher biomass on sandy silt than on the other two soil types (*p* < 0.018). Focusing on the dry mass changes of the edible parts caused by the iodine treatment, the 0.5 mg/L iodine concentration had a moderate stimulating (3–13%) and inhibiting effect (11–19%) on the biomass production of potato tubers and carrot roots, respectively. However, it must be emphasized that, based on statistical analysis, these differences were not significant (*p* > 0.062) and independent from the soil type; thus, the applied iodine treatments had neither a negative nor a positive effect on the yield of potato and carrot plants.

**TABLE 5:** Average photosynthetic efficiency and chlorophyll content index of potato leaf organs cultivated in three different soils and irrigated with water containing iodine.

Dry mass/g (*n* = 3; RSD%)						

		Potato			Carrot	
			
	Iodine concentration in irrigation water	Root	Aerial part	Tuber	Root	Aerial part
Sand	Control	2.42^a^(9)	9.72^a^(9)	38.1^a^(5)	17.0^a^(22)	13.5^a^(2)
	0.1 mg/L	3.41^a^(12)	9.50^a^(9)	36.0^a^(11)	17.0^a^(1)	12.1^a^(13)
	0.5 mg/L	3.26^a^(11)	10.0^a^(8)	43.2^a^(10)	13.8^a^(22)	12.0^a^(8)
Sandy silt	Control	2.29^a^(10)	8.98^a^(12)	36.7^a^(7)	25.5^a^(8)	13.8^a^(20)
	0.1 mg/L	2.22^a^(15)	8.44^a^(3)	39.0^a^(6)	21.6^a^(2)	11.6(4)a
	0.5 mg/L	2.04^a^(22)	8.37^a^(12)	37.8^a^(19)	21.8^a^(8)	16.0^a^(9)
Silt	Control	2.58^a^(12)	11.0^a^(20)	36.8^a^(1)	14.3^a^(8)	11.4^a^(9)
	0.1 mg/L	2.86^a^(8)	11.4^a^(8)	38.7(6)a	14.2^a^(12)	10.0^a^(10)
	0.5 mg/L	3.11^a^(8)	10.3^a^(5)	37.8^a^(22)	12.8^a^(15)	12.1^a^(12)

*Different letters indicate significant differences (p < 0.05, Tukey’s test) within the soil types.*

### Iodine Concentration and Distribution in Different Plant Parts

**FIGURE 1:**
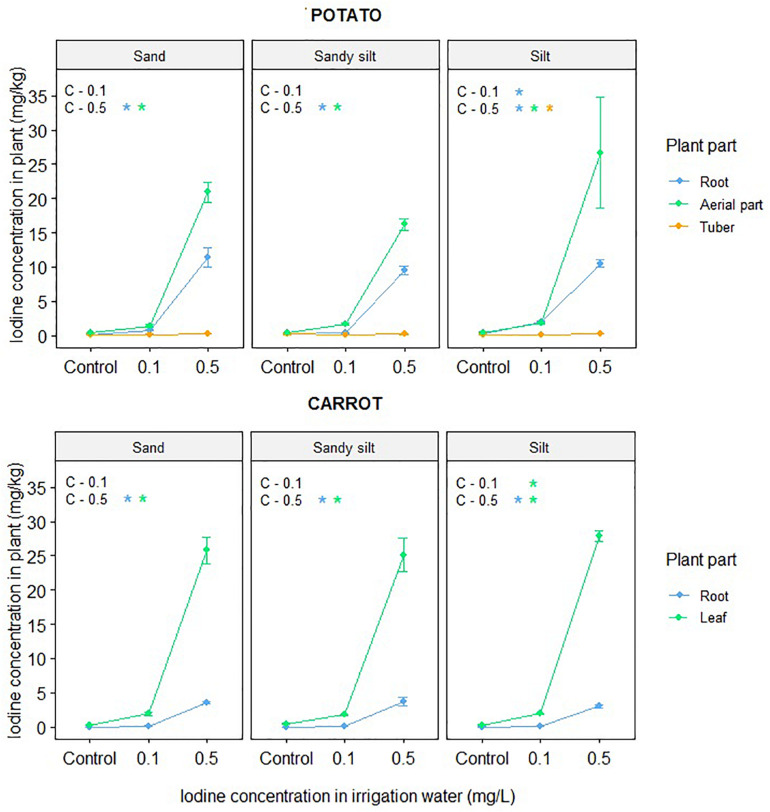
Iodine concentration in different parts of potato and carrot plants cultivated and irrigated with iodine-containing (0.1 and 0.5 mg/L) water in different soils (sand, sandy silt, and silt). Significant treatment effects are indicated on the top-left corner; *p < 0.05 based on linear regression.

The iodine concentrations in potato and carrot plants treated with irrigation water containing iodine at various concentrations and cultivated in different soils (sand, sandy silt, silt) are shown in Figure 1. In the control samples of potato tubers and carrot roots, the iodine concentrations varied in the range of 0.012–0.013 and 0.15–0.20 mg/kg, respectively, independently from the soil type (*p* > 0.2 for the soil effect). On applying higher iodine dosages in the irrigation water, the iodine concentration of different plant organs increased simultaneously, especially when the iodine dosage was adjusted to 0.5 mg/L (*p* = 0.03). For both plants, the highest iodine concentration was observed in the aerial parts and the lowest in carrot roots and potato tubers, although the iodine accumulation in carrot roots was of one order magnitude higher than in potato tubers. This difference in the reaction/sensitivity of the two plants’ edible parts to the treatment was also significant (*p* < 0.001). On applying the highest iodine dosage in irrigation water and cultivating the investigated plants in sand, sandy silt, and silt soil, the average iodine concentration in the edible parts were 0.21, 0.19, and 0.27 mg/kg DW (potato) and 3.5, 3.7, and 3.0 mg/kg DW (carrot), respectively. Based on these results, silt soil (in the case of potato tubers) and sand or sandy silt soil (for carrot) seem to be the best media for biofortification with iodine. Considering the dry weight and the iodine concentrations of the different plant tissues, the average iodine distribution among the different plant parts was calculated (Table 6). In potato control samples cultivated in different soils, these values amounted to 3–5% (root), 33–44% (aerial part), and 51–63% (tuber) and showed a moderate soil effect. However, in the case of carrot control plants, the iodine distribution ratios (5 to 6% root and 94–95% aerial part) were not influenced by the soil properties. On applying irrigation with iodine-containing water, the distribution of iodine within the plants changed drastically: the distribution was shifted from the tubers (potato) and aerial parts (carrot) to the roots, i.e., an opposite trend was observed for carrot roots and potato tubers, where at 0.5 mg/L iodine concentration the distribution values amounted to 10–17 and 3–4%, respectively. Based on the results, the carrot seems to be a promising candidate for biofortification with iodine.

**TABLE 6:** Average iodine distribution among different plant tissues of potato and carrot plants cultivated in different soils.

Iodine distribution (%)						

		Potato			Carrot	
			
	Iodine concentration in irrigation water	Root	Aerial part	Tuber	Root	Aerial part
Sand	Control	3^a^	39^a^	58^a^	6^a^	94^a^
	0.1 mg/L	12^b^	62^b^	26^b^	10^a,b^	90^a,b^
	0.5 mg/L	14^b^	82^c^	4^c^	14^b^	86^b^
Sandy silt	Control	4^a^	33^a^	63^a^	5^a^	95^a^
	0.1 mg/L	4^a^	68^b^	28^b^	12^b^	88^b^
	0.5 mg/L	12^b^	84^c^	4^c^	17^c^	83^c^
Silt	Control	5^a^	44^a^	51^a^	5^a^	95^a^
	0.1 mg/L	18^b^	65^b^	17^b^	7^a^	93^a^
	0.5 mg/L	10^a^	87^c^	3^c^	10^a^	90^a^

*Different letters indicate significant differences (p < 0.05, Tukey’s test) within the soil types, separated by plant parts.*

### Concentration of Selected Macro- and Microelements

The concentrations of the selected macro- and microelements in different plant parts of potato and carrot plants, cultivated on sand, sandy silt, and silt soils, are listed in Table 7. In the edible parts of potato and carrot plants, the concentrations of Mn, Cu, Zn, and B did not change significantly (*p* > 0.057), even at the highest iodine concentration of the irrigation water, while Mg, P, and Fe concentrations changed to varying degrees. In potato tubers, the Mg and P concentrations decreased significantly (*p* < 0.004), mainly independently of the soil properties (except the concentration of P, which significantly increased on sandy silt; *p* < 0.005), and the iodine treatment resulted in a significantly higher Fe accumulation (*p* < 0.004), except on silt soil, where there was no significant effect observed (*p* > 0.06). In turn, in carrot plants, the Mg and Fe accumulation of root samples cultivated on sandy silt was partly inhibited (*p* < 0.04); however, in the other two soils, the uptake of Mg, P, and Fe was stimulated by the iodine treatment (but it was significant only on silt soil).

**TABLE 7:** Average macro- and micronutrient concentrations of potato and carrot plant parts cultivated in three different soils and irrigated with water containing iodine.

	Sand				Sandy silt			Silt		
			
		Root	Aerial part	Tuber	Root	Aerial part	Tuber	Root	Aerial part	Tuber
**Potato**										
Mg (mg/kg)	Control	6,311175^a^	17,0672,224^a^	87464^a^	4,690848*a*	9,4141,484^a^	93398^a^	4,905557^a^	12,222962^a^	1,447404^a^
	0.1 mg/L	2,017152^b^	12,006536^b^	1388^b^	94162^b^	6,807244*a*^b^	31837^b^	2,598500^b^	10,616342^a,b^	20514^b^
	0.5 mg/L	2,458426^b^	11,430538^b^	22613^b^	1,467428^b^	6,2621,346^b^	434118^b^	2,419112^b^	9,771575^b^	30410^b^
P (mg/kg)	Control	1,017259^a^	1,338214^a^	1,566100^a^	1,12759^a^	1,864271^a^	1,59967*a*	1,19420*a*	1,650228^a^	3,146592^a^
	0.1 mg/L	47742^a^	1,073131^a^	1,207260^a,b^	56282^b^	1,40770^b^	2,203220^b^	77956^b^	1,386206^a^	1,29096^b^
	0.5 mg/L	60296^a^	1,02083^a^	1,11083^b^	98977*a*	1,44268^b^	2,28290^b^	1,03127^c^	1,596262^a^	1,31352^b^
Mn (mg/kg)	Control	377^a^	459^a^	41*a*	193*a*	4810^a^	41*a*	2711*a*	405^a^	62^a^
	0.1 mg/L	321^a^	3913^a^	41^a^	221^a,b^	462^a^	41^a^	302^a^	394^a^	41^a^
	0.5 mg/L	466^a^	417^a^	41^a^	328^b^	394^a^	51^b^	394^a^	323^a^	41^a^
Fe (mg/kg)	Control	55522^a^	9917^a^	181^a^	428102^a^	14917^a^	288^a^	907119^a^	11414^a^	331^a^
	0.1 mg/L	1,47181^b^	18216^b^	547^b^	1,05765^b^	24419^b^	525^b^	1,30948^b^	1999^b^	462^a^
	0.5 mg/L	1,53494^b^	17018^b^	476^b^	1,648262^c^	18642^a,b^	641^b^	1,97795^c^	1848^b^	388^a^
Cu (mg/kg)	Control	102^a^	51^a^	30.3^a^	92^a^	71^a^	40.5^a^	111^a^	61^a^	61^a^
	0.1 mg/L	122^a,b^	41^a,b^	30.3^a^	111^a^	51^a^	50.5^a^	123^a^	62^a^	41^a^
	0.5 mg/L	193^b^	61^a,c^	30.3^a^	237^b^	61^a^	60.6^a^	181^b^	61^a^	41^a^
Zn (mg/kg)	Control	17424^a^	371^a^	323^a^	20028^a^	543^a^	382^a^	16711^a^	454^a^	416^a^
	0.1 mg/L	1631^a^	394^a^	363^a^	20810^a^	524^a^	412^a^	1788^a^	474^a^	411^a^
	0.5 mg/L	1635^a^	4610^a^	361^a^	18910^a^	472^a^	435^a^	1764^a^	443^a^	403^a^
B (mg/kg)	Control	211^a^	526^a^	60.6^a^	271^a^	808^a^	70.7^a^	231^a^	6410^a^	60.6^a^
	0.1 mg/L	261^b^	555^a^	50.6^a^	262^a^	864^a^	60.6^a^	251^b^	663^a^	60.6^a^
	0.5 mg/L	261^b^	527^a^	60.5^a^	253^a^	709^a^	72^a^	261^b^	621^a^	70.6^a^
**Carrot**										
Mg (mg/kg)	Control	1,348353^a^	4,343476^a^		1,953202^a^	4,010821^a^		1,213215^a^	4,995517^a^	
	0.1 mg/L	1,831151^a^	5,544505^b^		1,56467^a,b^	6,259730^b^		1,637139^b^	4,623290^a^	
	0.5 mg/L	1,737293^a^	2,867343^c^		1,392207^b^	3,172152^a^		1,85587^b^	3,275139^b^	
P (mg/kg)	Control	2,187393^a^	3,544214^a^		2,516211^a^	3,777374^a^		1,79464^a^	3,248456^a^	
	0.1 mg/L	2,457479^a^	5,039221^b^		2,990457^a^	6,125579^b^		2,880261^b^	4,350186^b^	
	0.5 mg/L	3,076331^a^	3,155223^a^		2,99391^a^	3,097518^a^		2,458431^a,b^	1,890368^c^	
Mn (mg/kg)	Control	91^a^	573^a^		91^a^	485^a^		61^a^	713^a^	
	0.1 mg/L	111^a,b^	911^b^		101^a^	966^b^		101^b^	647^a^	
	0.5 mg/L	132^b^	474^c^		81^a^	352^c^		91^b^	363^b^	
Fe (mg/kg)	Control	571^a^	1219^a^		739^a^	13118^a^		5713^a^	12512^a^	
	0.1 mg/L	678^a^	17424^b^		562^b^	2446^b^		585^a^	16549^a^	
	0.5 mg/L	544^a^	9310^a^		575^b^	1014^c^		772^a^	1232^a^	
Cu (mg/kg)	Control	41^a^	41^a^		51^a^	51^a^		41^a^	51^a^	
	0.1 mg/L	91^b^	51^a^		81^b^	72^a,b^		81^b^	92^a,b^	
	0.5 mg/L	61^a^	41^a^		102^b^	41^a,c^		71^b^	51^a,c^	
Zn (mg/kg)	Control	264^a^	244^a^		377^a^	331^a^		333^a^	324^a^	
	0.1 mg/L	383^b^	131^b^		415^a^	345^a^		321^a^	345^a^	
	0.5 mg/L	221^a^	182^a,b^		353^a^	253^b^		311^a^	211^b^	
B (mg/kg)	Control	142^a^	282^a^		152^a^	327^a^		131^a^	283^a^	
	0.1 mg/L	135^a^	332^a^		151^a^	373^a^		161^a,b^	311^a^	
	0.5 mg/L	182^a^	315^a^		162^a^	315^a^		171^b^	282^a^	

*Letters indicate significant differences (p < 0.05, Tukey’s test) within soil types, separated by plant part.*

## Discussion

During the development of a biofortification technology based on irrigation with doped water, the aims are not only to achieve an increased concentration for the selected target element in the edible plant tissues but to also simultaneously study its effect on plant physiological processes, yield, and the microbiological community in the soil. Looking at the efficiency of biofortification with iodine calculated for the edible parts of potato and carrot plants, cultivated on three different soils and irrigated with KI doped water, it can be established that the iodine concentrations were increased by a factor of 1.1–1.7 and 232–308, respectively.

The applied biofortification technique had no significant effect on the iodine content of potato tubers grown on sand and sandy silt soils, but on silt soil, applying 0.5 mg/L iodine concentration, an increment was observed. Following the uptake, the largest part of iodine was translocated to the aerial part of potato plants, and only about 3 to 4% of the target element remained in the tubers. In the literature, only a few studies have focused on the biofortification of potato with iodine, and the applied dosages were in an order of magnitude higher than in our experiment. Using an iodine-containing fertilizer (250–5,000 g I/ha) or irrigation water (500–1,000 mg/L), the iodine accumulation in potato tubers amounted to 0.06–0.89 mg/kg FW (Caffagni et al., 2012) and 34 mg/kg FW (Caffagni et al., 2011), respectively. In another experiment where potato plants were cultivated in loess soil (pH = 8.16, OM = 1.36%, total iodine = 1.7 mg/kg), by applying fertilizer in a surface concentration of 0.59 kg I/ha, no change was observed in the iodine concentration as compared to the control samples (Mao et al., 2014), which is similar to our results.

On increasing the iodine concentration in irrigation water, the iodine accumulation in carrot roots increased simultaneously, especially when the dosage was shifted to 0.5 mg/L. The maximum iodine concentration of dried root samples cultivated on sandy silt and sand soil amounted to 3.7 and 3.5 mg/kg, respectively. In the case of silt soil, the iodine concentration of carrot roots was lower (3.0 mg/kg). This phenomenon can be explained by the fact that sand and sandy silt soils have lower organic matter content (Table 1); therefore, a lower amount of iodine is bound to humic and fulvic acids, and thus iodine mobility is higher (Medrano-Macías et al., 2016; Gonzali et al., 2017). In former experiments, fertilizers containing iodide at various concentrations (2–150 mg/kg) were applied for the biofortification of carrot plants grown on Inceptisol (pH = 5.91, OM = 4.09%, total iodine = 2.02 mg/kg; Hong et al., 2008) and Udic Luvisol (pH = 7.85, OM = 1.39%, total iodine = 0.88 mg/kg; Dai et al., 2004) soils. It was established that the maximum iodine content in carrot roots was 30 mg/kg FW (Hong et al., 2008) and 0.9 mg/kg DW (Dai et al., 2004). In a long-term (2 years) experiment, 2 and 5 kg I/ha surface concentration was applied, and the carrot plants were cultivated in silt loam (pH = 6.98–7.10, OM = 2.84–3.41%, total iodine was not given; Smoleń et al., 2011) and silty clay (pH = 6.10–7.77, OM = 2.11–2.48%, total iodine = 0.24–0.25 mg/kg; Smoleń et al., 2016b) soil, and the concentration in the carrot roots amounted to 4.3 mg/kg DW (Smoleń et al., 2011) and 25 mg/kg DW (Smoleń et al., 2016b), respectively.

Chlorophyll-a fluorescence and the photosynthetic pigment content of leaves are considered as sensitive parameters that indicate well the stress-induced changes in the photosynthetic apparatus (Ahammed et al., 2018). Comparing the Fv/Fm ratios and CCI values, it can be established that the applied iodine treatment had no significant effect on these parameters; however, the effect of soil properties on the maximum quantum efficiency of PSII and chlorophyll content index was detectable, resulting in higher values in case of silt soil compared to sand and sandy silt. The highest CCI values in silt soils could be caused by the higher concentrations of all N-forms in it. A close correlation has been reported between leaf chlorophyll content and plant nitrogen status in many agricultural crops (Liu et al., 2019; Padilla et al., 2019). As there is no literature data on the effect of iodine treatment on the photosynthetic efficiency of potato plants, it is worth mentioning that, in our former experiments with the same experimental setup, we found the same results for tomato plants (which belong to the same *Solanaceae* plant family as potato), too. However, considering the effect of soil, in tomato plants, the physiological parameters were the lowest on silt soil (Dobosy et al., 2020b).

Based on our results, we can conclude that the iodine treatment had no significant effect on dry mass production in the edible organs of potato and carrot plants. These observations are partly in line with the literature data; however, it has to be noted that the treatment technologies and the applied iodine concentrations were different. For example, in the case of potato, an inhibitor effect (85%) was observed at a high surface concentration (5 kg I^–^/ha) of the fertilizer (Caffagni et al., 2011), but no significant effect was observed while using fertilizer in the concentration of 0.59 g I^–^/ha (Mao et al., 2014). At the iodine concentration of 2 kg I/ha (Smoleń et al., 2014), 5 kg I/ha (Smoleń et al., 2016b), 5 mg/kg soil (Dai et al., 2004), and 500 mg I/L (Signore et al., 2018), the biofortification process did not influence the growth of carrot plants. Another study reported that using 150 mg I/kg fertilizer decreased the dry mass of carrot plants by 18% (Hong et al., 2008).

In the literature, there is not any experimental data focusing on the effect of iodine on the macro- and microelement content of potato plants, while for carrots there is only one (Smoleń et al., 2011). Our observations harmonize with these earlier published data that the iodine treatment resulted in higher accumulation in carrot roots cultivated in silt loam soil. In our experiment, depending on the P content of sand, sandy silt, and silt soils, the phosphorous concentration increased in the carrot roots compared to the control samples by about 20, 40, and 60%, respectively. It should be noted that the silt soil had the highest CEC, organic matter, and total nitrogen concentrations; however, the complex effect of these physico-chemical properties are presently unknown.

Comparing the concentration of P, Mg, and Fe in tomato, cabbage (Dobosy et al., 2020b), carrot, and potato cultivated at the same growing conditions, it can be established that the iodine had a different effect on the accumulation of these elements in their edible parts. Due to the various soil quality and the different uptake and translocation processes of the investigated plants, the concentration of P, Mg, and Fe related to the control plants changed in the following ranges: −58 + 43, −79 + 53, and −73 + 161%, respectively. It means that the iodine treatment resulted in the highest influence on the iron household of these plants.

## Conclusion

This study demonstrated the biofortification of potato and carrot with iodine and the effect of iodine on the photosynthetic processes, dry mass production, as well as on selected macro- and microelement concentrations of these plants cultivated on sand, sandy silt, and silt soils. Our results show that the iodine content of potato tubers cannot be increased significantly; therefore, this plant is not suitable for the biofortification of iodine. However, carrot plants have much higher iodine accumulation capacity, independently of the properties of soils used for cultivation, i.e., in their roots, the iodine concentration can be increased by one order of magnitude by applying irrigation with 0.5 mg/L iodide-containing water. Considering the iodine concentration values and the moisture content of carrot roots, it can be calculated that consuming 100 g fresh carrot would be enough to cover about 38% of the daily iodine intake requirement for an average adult person.

## Data Availability Statement

The raw data supporting the conclusions of this article will be made available by the authors, without undue reservation.

## Author Contributions

PD was the leader of the project, performed the analytical experimental work, conducted the analysis of results, and prepared the manuscript. AE conducted the statistical data analyses and data visualization. VV participated in the analytical experimental work and data visualization. SS was involved in conducting the field experiment and in sample preparation. MR carried out the plant growing. TT carried out the photosynthetic efficiency and chlorophyll content measurements. GyZ provided substantial contributions to the conception of the work and gave final approval of the version to be published. All authors contributed to the article and approved the submitted version.

## Conflict of Interest

The authors declare that the research was conducted in the absence of any commercial or financial relationships that could be construed as a potential conflict of interest.
